# Multiple neoplasia in a patient with Gitelman syndrome harboring germline monoallelic *MUTYH* mutation

**DOI:** 10.1038/s41525-020-00146-9

**Published:** 2020-09-18

**Authors:** Jason Yongsheng Chan, Ming Ren Toh, Siao Ting Chong, Nur Diana Binte Ishak, Arun Mouli Kolinjivadi, Sock Hoai Chan, Elizabeth Lee, Arnoud Boot, Li Shao-Tzu, Min-Hoe Chew, Joanne Ngeow

**Affiliations:** 1 Division of Medical Oncology, National Cancer Centre Singapore, Singapore, Singapore; 2 SingHealth Duke-NUS Blood Cancer Centre, Singapore, Singapore; 3 Cancer Science Institute of Singapore, National University of Singapore, Singapore, Singapore; 4 Cancer Genetics Service, Division of Medical Oncology, National Cancer Centre Singapore, Singapore, Singapore; 5 Lee Kong Chian School of Medicine, Nanyang Technological University, Singapore, Singapore; 6 Centre for Computational Biology, Duke-NUS Medical School, Singapore, Singapore; 7 Programme in Cancer and Stem Cell Biology, Duke-NUS Medical School, Singapore, Singapore; 8 Department of General Surgery, Sengkang Health, Singapore, Singapore; 9 NUS Graduate School for Integrative Sciences and Engineering, Singapore, Singapore; 10 Oncology Academic Clinical Program, Duke-NUS Medical School, Singapore, Singapore; 11 Institute of Molecular and Cellular Biology, ASTAR, Singapore, Singapore

**Keywords:** Genetics research, Cancer genetics

## Abstract

Gitelman syndrome is a rare, recessively inherited disease characterized by chronic hypokalemia and hypomagnesemia as a result of defective electrolyte co-transport at the level of the distal convoluted tubule of the kidney. Here, we present the first report of a patient with Gitelman syndrome who developed multiple neoplasia including colorectal polyposis, synchronous colorectal cancers, recurrent breast fibroadenomata and a desmoid tumor. Whole-exome sequencing confirmed germline compound heterozygous mutations of c.179C > T and c.1326C > G in *SLC12A3*, and in addition, identified a monoallelic germline c.934-2A > G splice site mutation in *MUTYH*. *In vitro*, magnesium deficiency potentiated oxidative DNA damage in lymphoblastoid cell lines derived from the same patient. We postulate that monoallelic *MUTYH* mutations may manifest in the presence of cooperative non-genetic mechanisms, in this case possibly magnesium deficiency from Gitelman syndrome.

## Introduction

Gitelman syndrome is a rare, recessively inherited disease with a prevalence of 1 in 40,000 (OMIM #263800). This condition is characterized by metabolic alkalosis, hypokalemia, and hypomagnesemia as a result of defective electrolyte co-transport at the level of the distal convoluted tubule of the kidney. Most cases are linked to inactivating mutations of *SLC12A3* resulting in functional loss of the thiazide-sensitive sodium-chloride symporter^1^. To date, there has been no reported association of any cancer type with Gitelman syndrome. Recently, however, we came across an unusual case of a patient with Gitelman syndrome and carrying a germline monoallelic *MUTYH* c.934-2A > G variant, who subsequently developed multiple neoplasia including colorectal polyposis, synchronous colorectal cancers, recurrent breast fibroadenomas and a desmoid tumor.

*MUTYH* encodes for a DNA glycosylase responsible for the restoration of oxidative base damage via the base excision repair (BER) pathway. During DNA replication, an intact pathway prevents 8-oxoguanine-induced G:C > T:A transversion mutagenesis by excising mis-incorporated adenine^2^. Whilst biallelic *MUTYH* mutants result in impaired glycosylase function and invariably lead to a recessively inherited colorectal polyposis syndrome with a predisposition to colorectal cancer^3^, whether monoallelic carriers harbor a higher risk of cancers compared to the general population remains controversial^4^.

In this paper, we hypothesize and provide evidence that chronic magnesium deficiency from Gitelman syndrome may have potentiated oxidative DNA damage and enabled the phenotypic manifestation of monoallelic *MUTYH* mutation in this patient.

## Results

### Clinical case report

**Fig. 1:**
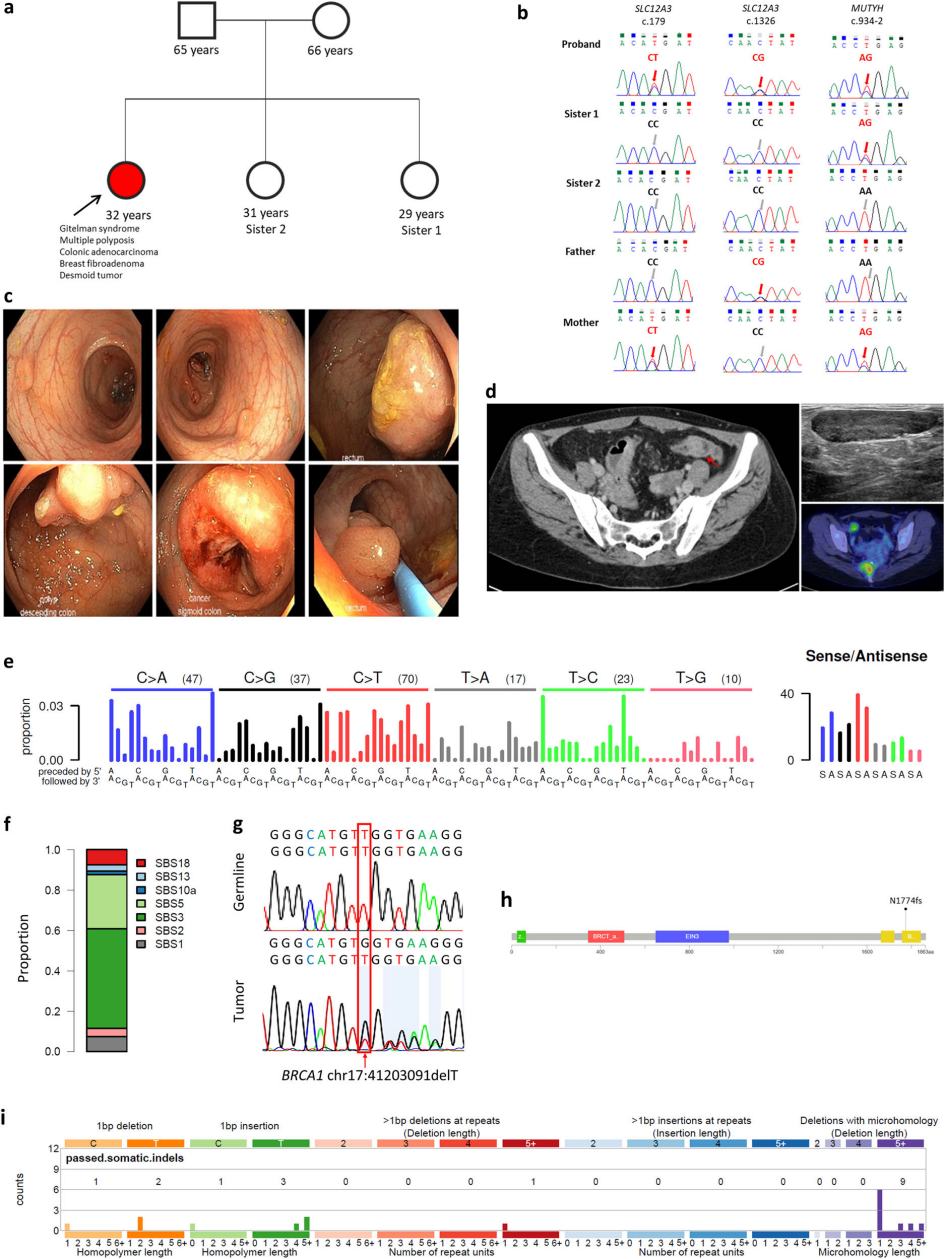
Clinical manifestations of the index patient. **a** Diagnosed with Gitelman syndrome at the age of 9 years, followed by multiple polyposis and colorectal carcinomas at age of 29 years. **b** Germline sequencing revealed compound heterozygous mutations of c.179C>T and c.1326C>G in *SLC12A3*, as well as a monoallelic germline c.934-2A>G splice site mutation in *MUTYH*. The corresponding genetic sequences for the rest of her family members are as shown. **c** Colonoscopy revealed the presence of multiple polyps, as well as synchronous tumors in the colorectum. **d** Images of sigmoid tumor on CT, breast fibroadenoma on ultrasound, and pre-sacral desmoid tumor on 18F-FDG PET/CT. **e** Mutational signature of the colorectal carcinoma of the index patient. **f** Proportions of mutations contributed by each inferred mutational signature are as shown. COSMIC signature 18 is attributed to DNA damage by reactive oxygen species, while signature 3 is due to defective homologous recombination-based DNA damage repair. **g**, **h** Somatic frameshift deletion mutation at exon 20 of the *BRCA1* gene. **i**) Indel mutational spectrum showing that the majority of indels were ≥5bp deletions, with microhomology, which is consistent with *BRCA1* inactivating mutation leading to defective homologous recombination-based DNA damage repair.

The index patient was a 29-year-old Chinese woman from Singapore who first presented with intermittent bleeding per rectum over a 3-month duration. Her medical record was significant for a history of Gitelman syndrome diagnosed at the age of 9 years, for which she has been prescribed long-term electrolyte supplements, indometacin, and spironolactone. Notably, due to non-compliance to medication, she was repeatedly admitted (up to eight times per year) for symptomatic electrolyte deficiency, each time with carpopedal spasms, peri-oral paraesthesia and generalized weakness. At the age of 24 years, she developed a left breast juvenile fibroadenoma, which was surgically resected. She was a never smoker and teetotaller. She was the eldest amongst three daughters of non-consanguineous parents and no significant family history was noted (Fig. 1a).

Initial physical examination was unremarkable except for pallor. Laboratory investigations demonstrated iron deficiency anemia (hemoglobin 8.1 g/dL, serum iron 2 µmol/L, ferritin 14.4 µg/L, total iron-binding capacity 68 µmol/L, transferrin saturation 2.9%), and hypokalemia (potassium 2.7 mmol/L). Urinary studies were significant for hyperkaluria (potassium 160 mmol/day) and hypermagnesuria (magnesium 6.96 mmol/day). Estimated creatinine clearance by Cockcroft-Gault equation was 60 ml/min. A whole-body CT-scan revealed a focus of eccentric mural thickening at the sigmoid colon as well as a few prominent small volume nodes in the sigmoid mesentery, with no evidence of distant metastatic disease. Colonoscopy showed the presence of multiple small sessile polyps scattered throughout the descending/sigmoid colon and rectum with no polyps seen on the right colon. A 3 cm sessile half-circumferential dysplastic tumor in the descending colon and another circumferential malignant-appearing lesion in the sigmoid colon were seen. A 3 cm sessile polyp was seen in the proximal rectum and a smaller pedunculated polyp was seen in the low rectum (Fig. 1c).

The patient underwent ultra-low anterior resection and creation of an end colostomy. Two synchronous moderately differentiated invasive adenocarcinomata in the sigmoid colon (pT3) and rectosigmoid junction (pT2) were histologically verified, with the latter arising in the background of a tubulovillous adenoma. Five out of 43 lymph nodes were positive for metastatic carcinoma (pN2a). Multiple tubular adenomata and a tubulovillous adenoma with high and low-grade dysplasia were identified. Despite the prevalence of multiple adenomas and synchronous tumors, it was decided by the surgical team after discussion with the patient and her family, that a panproctocolectomy with end ileostomy or pouch anastomosis with a temporary ileostomy would render a high risk of life-threatening hypokalemia. The decision for permanent colostomy rather than performing a low colorectal anastomosis as a temporary ileostomy with its concurrent risks of life-threatening hypokalemia, would be required.

Following the diagnosis of stage IIIB synchronous colon cancers, adjuvant chemotherapy was commenced. Due to concerns of chemo-related toxicity potentiating electrolyte losses, she was started on biweekly infusional 5-fluorouracil following the simplified De Gramont regimen. She tolerated the initial treatment well and oxaliplatin was added from the third cycle onwards following the mFOLFOX6 regimen and was continued for 4 cycles. The treatment course was challenging as she required increasingly frequent intravenous replacements of potassium chloride for progressive severe hypokalemia. After her 6th cycle of chemotherapy, she required inpatient telemetry monitoring for severe hypokalemia associated with U-waves on electrocardiography. The decision was made to permanently discontinue chemotherapy in view of grade 4 toxicity (CTCAE v4.0). The patient was followed up with weekly evaluations of serum potassium levels up till 3 months from chemotherapy. Surveillance imaging 18 months later revealed a 0.8 cm nodule in her left breast, as well as a 4 cm pre-sacral soft tissue mass (Fig. 1d). Needle biopsies confirmed a recurrent breast fibroadenoma and de novo desmoid tumor, respectively. She otherwise remained in remission at 42 months from diagnosis.

### Genetic workup by whole-exome sequencing

Whole-exome sequencing using DNA isolated from peripheral blood and the colonic adenocarcinoma revealed germline compound heterozygous mutations of c.179C > T and c.1326C > G in *SLC12A3*, consistent with her clinical diagnosis of Gitelman syndrome. The c.179C > T variant was maternally inherited, while the c.1326C > G variant was paternally inherited. In addition, we identified a monoallelic germline c.934-2A > G splice site mutation in *MUTYH* as a potentially relevant pathogenic variant contributing to her clinical syndrome of colonic polyposis and synchronous adenocarcinomata (Supplementary Tables 1 and 2). These mutations were validated by Sanger sequencing (Fig. 1b). Germline mutations in other known cancer susceptibility genes, such as *TP53* and *APC*, were not identified. Germline sequencing of her family members confirmed the presence of heterozygous *MUTYH* c.934-2A > G splice site variant in Sister 1 and her mother, while her father was homozygous wild-type, consistent with a dominant pattern of inheritance.

In the tumor sample, no additional somatic mutations of *MUTYH* or loss of heterozygosity at its genomic locus were observed. Monoallelic somatic nonsynonymous mutations in *PIK3CA* c.2046G > C as well as truncating mutations of *TP53* c.154C > T and *STAT4* c.1085C > A were identified. Further testing of the tumor samples did not show evidence of microsatellite instability or somatic mutations in *KRAS*, *NRAS*, and *BRAF* by targeted sequencing. The estimated tumor mutation burden was consistent with a modest mutator phenotype at 6.8 mutations per megabase. Analysis of tumor mutational signatures revealed that COSMIC signature 3 was the dominant signature (Fig. 1e, f). This signature, attributed to defective homologous recombination-based DNA damage repair, is supported by our observation of a pathogenic somatic frameshift deletion-T mutation at exon 20 of the *BRCA1* gene, as well as an indel mutational spectrum showing that the majority of indels were ≥ 5 bp deletions with microhomology (Fig. 1g–i). Another notable mutational pattern identified was signature 18 (oxidative DNA damage by reactive oxygen species (ROS)), which has been reported in *MUTYH*-deficient cancers^5–8^.

### Enhancement of oxidative DNA damage by magnesium deficiency

**Fig. 2:**
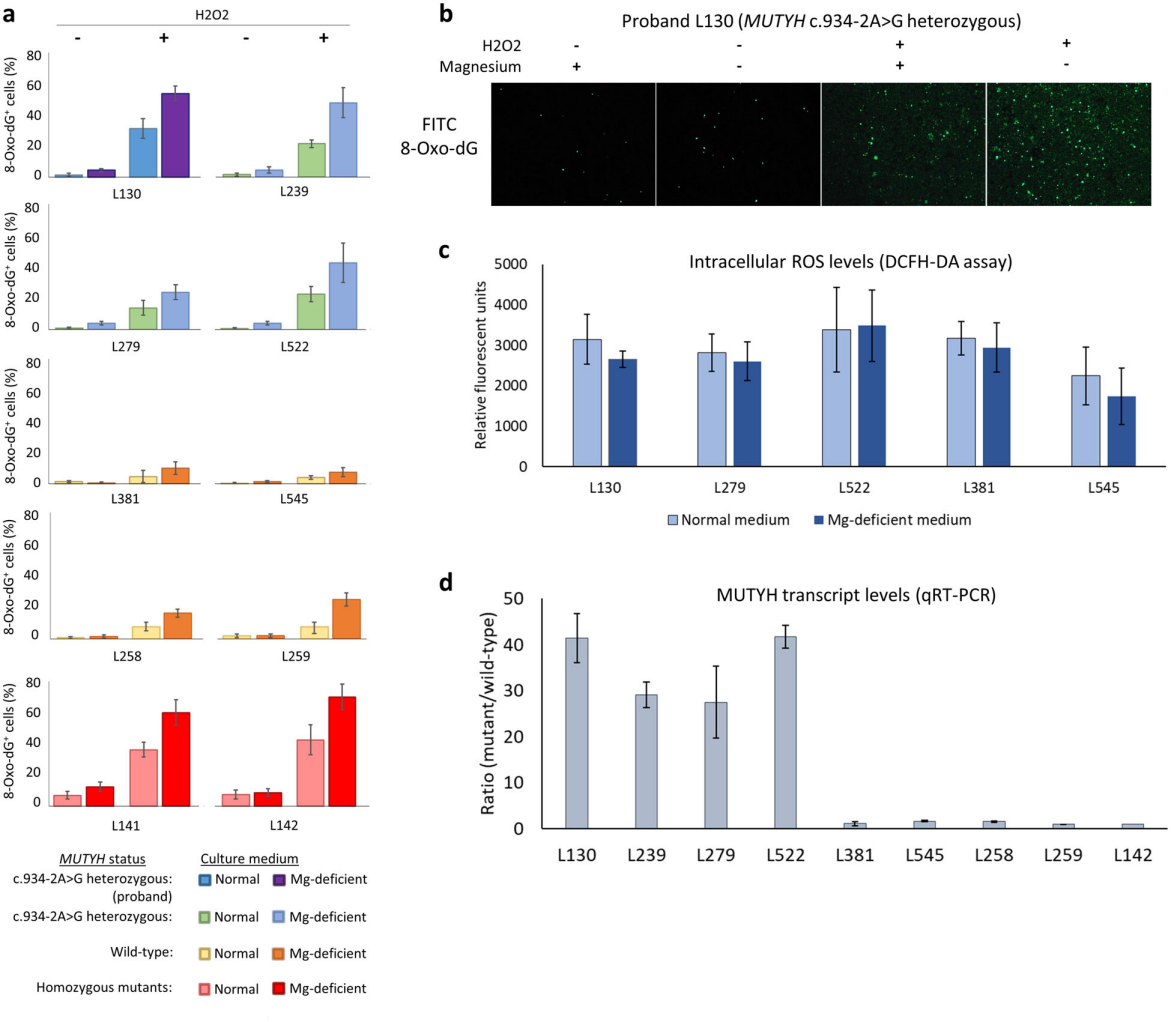
Susceptibility of *MUTYH* variants to oxidative DNA damage in magnesium deficiency. **a** When exposed to oxidative stress (hydrogen peroxide 200 µM, 16 h), cell lines with monoallelic *MUTYH* c.934-2A>G variants displayed markedly greater levels of DNA damage when cultured in magnesium-deficient media (mean: 22.2% vs 41.9%; *p* = 0.037), in a similar trend to those with homozygous *MUTYH* mutations (mean: 39.5% vs 65.0%; *p* = 0.049). **b** Representative images showing the level of DNA damage (FITC signals) for proband sample L130. **c** Intracellular reactive oxygen species (ROS) levels were comparable in cells cultured in normal and magnesium-deficient media. **d** *MUTYH* c.934-2A > G variant alleles in heterozygous carriers (L130, L279, L522, and L239) were selectively expressed over wild-type alleles. All experiments were performed in triplicate and results presented as mean ± standard deviation.

We obtained EBV-immortalized B lymphoblastoid cell lines derived from the proband, her family members, and other individuals with germline *MUTYH* variants. At baseline, spontaneous oxidative DNA damage as measured by percentage of 8-oxo-2’-deoxyguanosine (8-oxo-dg) positive cells were lower in cell lines harboring homozygous wild-type alleles or monoallelic *MUTYH* c.934-2A > G variants, as compared to cell lines with homozygous mutants (mean: 1.1%, 1.1% vs. 7.3%, respectively; *p* < 0.001). Culture in magnesium-deficient media resulted in mild elevation in DNA damage levels in the cell lines, particularly in monoallelic *MUTYH* c.934-2A > G variants (mean: 1.1% vs. 4.3%; *p* < 0.001) (Fig. 2a, b). Intracellular ROS levels were comparable in cells cultured in normal and magnesium-deficient media, suggesting that DNA damage in the context of magnesium-deficiency is due to defective DNA repair machinery rather than increased ROS production (Fig. 2c). Moreover, when exposed to oxidative stress (hydrogen peroxide 200 µM, 16 h), cell lines with monoallelic *MUTYH* c.934-2A > G variants displayed markedly greater levels of DNA damage when cultured in magnesium-deficient media (mean: 22.2% vs 41.9%; *p* = 0.037), in a similar trend to those with homozygous *MUTYH* mutations (mean: 39.5% vs 65.0%; *p* = 0.049). Supporting their functional significance, *MUTYH* c.934-2A > G variant alleles in heterozygous carriers (L130, L239, L279, L522) were selectively expressed over wild-type alleles. As a control, the variant allele was not detected in samples known to be wild-type at this locus (L381, L545, L258, L259, and L142) (Fig. 2d). The wild-type allele was not detected in sample L141 (data not shown). No differences in cell viability were noted in the cell lines grown with or without supplemented magnesium (Supplementary Fig. 1).

## Discussion

Recent studies on patient-derived lymphoblastoid cell lines showed that cells harboring monoallelic p.R245H *MUTYH* variants exhibited mutator phenotypes intermediate between wild-type and homozygous cells (suggesting a gene dosage effect), while p.Y179C carriers were similar to homozygous cells (suggesting a dominant-negative effect)^9^. In our patient with young-onset synchronous colorectal cancer on a background of multiple colonic adenomatous polyposis, we identified a potentially pathogenic germline *MUTYH* c.934-2A > G splice acceptor site mutation, consisting of an A > G substitution at the −2 position of intron 10. This variant has been reported to produce an aberrant mRNA transcript leading to a premature stop codon. The resulting truncated MYH protein lacks a nuclear localization signal and is excluded from the nucleus, suggesting that BER may be impaired^10^. In keeping with this, we observed that *MUTYH* c.934-2A > G variant alleles in heterozygous carriers (L130, L279, L522, and L239) were selectively expressed over wild-type alleles, suggesting that most of the MUTYH protein expressed may be defective. Notably, however, although heterozygous c.934-2A > G has been reported in several individuals with colorectal adenomas and carcinomas^11–14^, most variant carriers—estimated at an allele frequency of 1.4% in East Asians on the ExAC database, do not harbour an increased risk of developing cancers over the general population. This negates any clinically significant gene dosage or dominant-negative effect exerted by this variant, and suggests that additional “hits” must occur to confer additional risk. Somatic inactivation of the wild-type copy of *MUTYH* in germline heterozygous carriers, while providing one possible explanation^8,10^, was not detected in our patient.

To our best knowledge, there have been no reported association with any cancer type with Gitelman syndrome in the existing literature, and our finding of a concurrent pathogenic *MUTYH* mutation led us to speculate that cooperative mechanisms account for the development of young-onset multiple neoplasia in our patient. Specifically, we hypothesized that the phenotypic consequences of Gitelman syndrome, specifically hypomagnesemia, may have contributed as a second “hit” leading to genetic instability and clinical manifestation of *MUTYH* deficiency^15^. Since magnesium is an essential cofactor in major enzymatic systems involved in DNA replication and repair^16^, and is an absolute requirement for downstream execution of BER pathways^17^, chronic magnesium deficiency due to Gitelman syndrome could possibly further abrogate BER efficiency in the setting of *MUTYH* deficiency, increasing susceptibility to oxidative stress and enabling the phenotypic manifestation of monoallelic *MUTYH* mutation in this patient. The analysis of mutational signatures lends support to this putative model of oncogenesis—the presence of COSMIC signature 18 due to oxidative DNA damage suggests a defective BER machinery^7^, while the larger contribution by COSMIC signature 3 may suggest that the somatic *BRCA1* mutation could have played a more dominant role downstream in the tumorigenic process. Taken together and from a larger healthcare perspective, it is tempting to speculate that magnesium deficiencies due to other intrinsic (such as polymorphisms of genes involved in magnesium cellular transport) or extrinsic causes (such as use of diuretics or proton pump inhibitors) may be of particular relevance to individuals with otherwise non-observable germline defects in DNA repair genes^16^.

In summary, this report highlights the potential pathobiological relevance of germline monoallelic *MUTYH* mutations in the presence of cooperative non-genetic mechanisms, in this case possibly chronic magnesium deficiency from Gitelman syndrome.

## Methods

### Patient data and specimens

All clinical information was retrieved from electronic medical records. Demographic data including sex, age, and ethnicity of the affected patient and her family members were verified against their National Registry Identification Cards. All histological parameters were reviewed by expert pathologists. EBV-immortalized B lymphoblastoid cell lines were derived from the proband (L130), her mother (L522), father (L381), sister 1 (L279) ,and sister 2 (L545). Lymphoblastoid cell lines from an unrelated individual with heterozygous *MUTYH* c.934-2A > G variant (L239), individuals harboring homozygous mutant *MUTYH* (L141 and L142), as well as healthy donors (L258 and L259) were also available (Supplementary Table 3). All cell lines were maintained in RPMI medium supplemented with 20% fetal bovine serum, 1% penicillin/streptomycin and 2 mM L-glutamine, with or without magnesium. Estimated magnesium concentration in complete medium was 1 mM (approximating physiological serum concentration), while magnesium-deficient medium contained ~0.2 mM. These cells were grown in a humidified chamber with 5% CO_2_ at 37 °C and maintained for at least 2 weeks before downstream experiments. Written informed consent from participants for the use of biospecimens and clinical data were obtained in accordance with the Declaration of Helsinki. Tissue collection and consent protocols were under ethics approval from the SingHealth Centralized Institution Review Board.

### Genomic DNA extraction

Genomic DNA from snap-frozen tumor tissue and peripheral whole blood samples were available from the index patient. Genomic DNA from her father and sister were obtained from peripheral blood samples. No material was available from her mother and other sister. Genomic DNA was extracted from tissue and blood specimens using the Blood & Cell Culture DNA Kit (Qiagen, Hilden, Germany). Genomic DNA yield and quality were determined by Quant-i PicoGreen dsDNA Assay Kit (Invitrogen, Carlsbad, CA, USA) and Nanodrop 1000 spectrophotometer (Thermo Scientific, Wilmington, DE, USA), and visually inspected by agarose gel electrophoresis.

### Library construction, whole-exome sequencing and bioinformatic analyses

Somatic mutations were identified by the Strelka2 variant caller with default parameters^18^. Variants were subsequently annotated by wAnnovar^19^. Tumor mutation burden was estimated based on the proportion of nonsynonymous single nucleotide variants and short indels per coding megabase. Mutational signature analysis was performed with mSigAct v2.0.0.9018^20^. All mutational signatures detected previously in whole-exome sequencing data were considered for initial mutational signature assignment using the SparseAssignActivity function, with a p.thresh at 0.5 to reduce stringency^21^. Mutational signature assignment was done after adjusting the signatures for exome trinucleotide abundance. To validate variants by Sanger sequencing, PCR amplicons encompassing the mutation sites were sequenced using the ABI PRISM BigDye Terminator Cycle Sequencing Ready Reaction kit (Applied Biosystems, Foster City, CA, USA) on an ABI 3730 xl DNA Analyzer (Applied Biosystems). Functional impact of variants were predicted using published tools^22–24^.

### Quantification of cell viability

Cell viability was quantified using the CellTiter-Glo 2.0 Luminescent Cell Viability Assay (Promega, USA), following the manufacturer’s protocol. In brief, were seeded at 2000 cells per 100 μl per well into 96-well plates. At 0, 24, 48, 72, and 96 h, the cells were incubated with CellTiter-Glo® 2.0 Reagent before absorbances were measured using a 96-well plate reader (Tecan M200 Infinite, Switzerland). For each time point, cell viability was evaluated as percentage of control absorbance at 0 h. All reactions were performed in triplicate and results presented as mean ± standard deviation.

### Immunofluorescence

DNA damage was visualized and quantified by direct counting of fluorescent cells by immunofluorescent microscopy. In brief, cells were seeded at 1000 cells per well in 8-well chamber slides (Ibidi GmbH, Martinsried, Germany) and incubated overnight. Cells were centrifuged onto the coverslip, washed once with PBS and then fixed in 100% methanol at −20 °C for 10 min. The fixed cells were blocked in blocking buffer (1% BSA and 0.05% Tween-20 in PBS) for 1 h, followed by three washes with PBS. The cells were then incubated with Anti-DNA/RNA antibody [15A3] 1:400 at 4 °C overnight (Abcam, Cambridge, UK), FITC-conjugated secondary antibody 1:800 at room temperature for 1 h (Santa Cruz Biotechnology, CA, USA), washed three times with PBS, before mounting with UltraCruz Mounting Medium (Santa Cruz Biotechnology, CA, USA). The same assay was performed following exposure to oxidative stress by treating the cells with hydrogen peroxide (ICM Pharma, Singapore) at 200 µM for 16 h.

#### RNA isolation and quantitative RT-PCR

Total RNA was extracted using the RNeasy Mini Kit according to the manufacturer’s protocol (Qiagen, Valencia, CA, USA) and subsequently treated with RQ1 RNase-free DNase (Promega, Madison, WI, USA). One microgram of DNase-treated RNA was reverse transcribed into cDNA using random primers and High-capacity cDNA Reverse Transcription Kit (Thermo Fisher Scientific, Waltham, MA, USA). Real-time PCR was performed on a CFX96 Touch™ (Bio-Rad Laboratories, Inc., Hercules, CA) using SsoFast EvaGreen Supermix (Bio-Rad Laboratories) with the following conditions: 95 °C for 30 s; followed by 40 cycles of 95 °C for 30 s and 60 °C for 5 s. Glyceraldehyde 3-phosphate dehydrogenase (*GAPDH*) was used as an endogenous control. The 2-ΔΔCt method was used to calculate fold changes in gene expression. Each sample was run in triplicate, and at least two experiments were analyzed. The following primer sequences were used: *MUTYH* wild-type transcript (forward 5′-AGC AGC TCT GGG GTC TAG CC-3′; reverse 5′ -TTC CTG CTC CAC TCT CTG GC-3′); *MUTYH* mutant transcript (forward 5′ -GGA AGG GGC AGT GAG AAG TC-3′; reverse 5′ -CCA GTG TTG GGA GCA CAC TC-3′) and *GAPDH* (forward 5′-GAAGGTGAAGGTCGGAGTCA-3′; reverse 5′-TTGAGGTCAATGAAGGGGTC-3′)^10^.

#### Cellular ROS assay

The measurement of ROS was performed using 2′,7′-Dichlorofluorescin Diacetate (DCFH-DA) (Sigma-Aldrich, St. Louis, MO, USA). The cells were washed with PBS, resuspended in 5 μM DCFH-DA staining solution and incubated for 30 min at 37 °C. The cells were washed with PBS and transferred to a 96-well black-coated microplate. Fluorescence intensity was measured using microplate reader at excitation 485 nm and emission 535 nm.

### Statistical analyses

Statistical analysis of mean values was performed through *t*-tests. All statistical analyses were conducted assuming a two-sided test with a significance level of 0.05 unless otherwise stated, and performed using MedCalc for Windows, version 18.2.1 (MedCalc Software, Ostend, Belgium).

### Reporting summary

Further information on research design is available in the Nature Research Reporting Summary linked to this article.

## Supplementary information

Supplementary Information

Reporting Summary

## Data Availability

The datasets used and/or analyzed in the current study are available from the corresponding author upon reasonable request. Whole-exome sequencing data for the patient are deposited in the European Genome-phenome Archive (EGA, accession number EGAS00001004609) and are available subject to Data Access Committee (DAC) approval.
