# Supplementary material for: Multiple neoplasia in a patient with Gitelman syndrome harboring germline monoallelic MUTYH mutation
Source: NPJ Genom Med. 2020 Sep 18;5:39. doi: 10.1038/s41525-020-00146-9 (PMC7501863; doi:10.1038/s41525-020-00146-9)
Supplement: Supplementary file 1 — Supplementary Information [file 41525_2020_146_MOESM1_ESM.pdf]

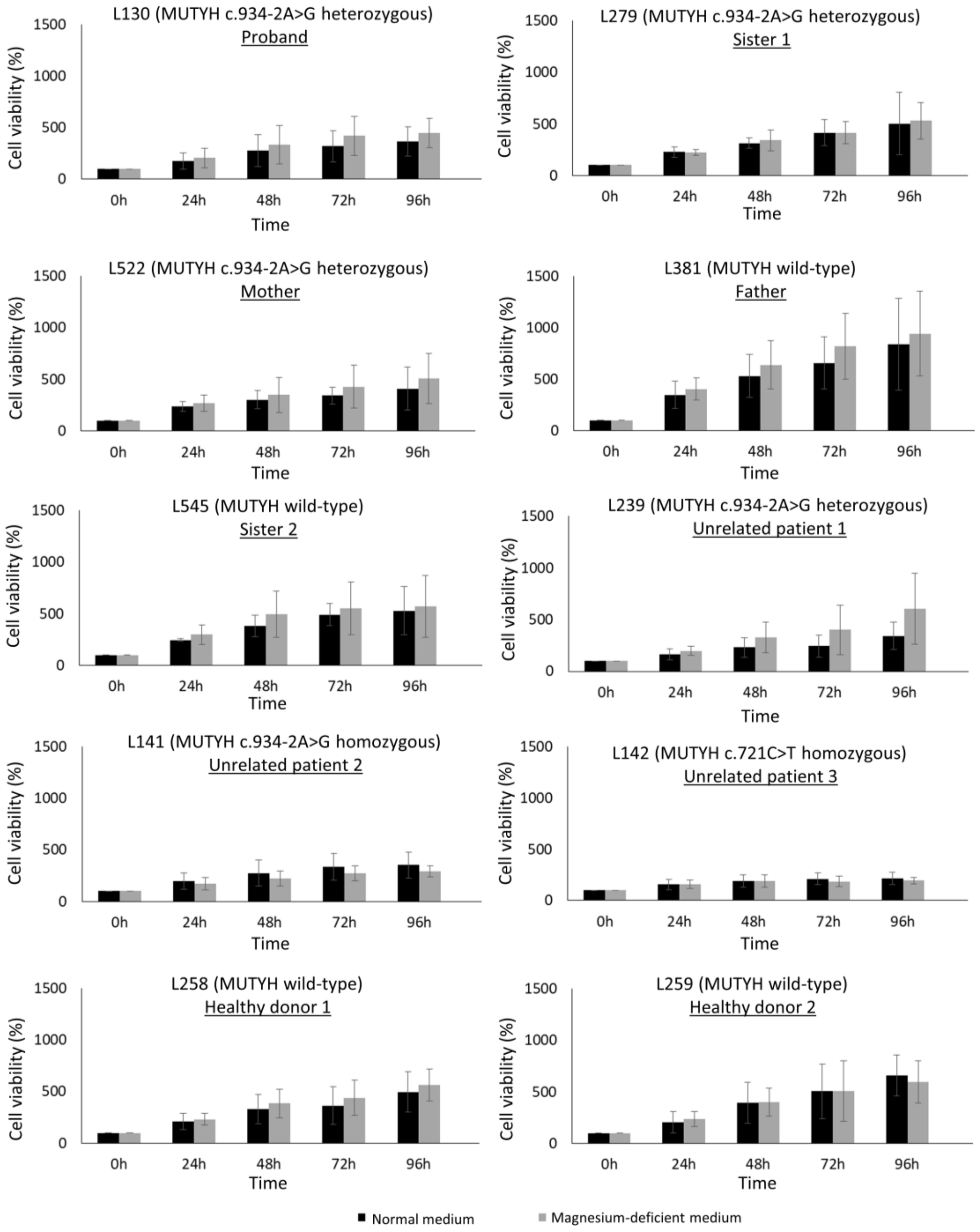

Supplementary Figure 1. EBV-immortalized B lymphoblastoid cell lines derived from patients. Base line cell viability of EBV-immortalized B lymphoblastoid cell lines harboring heterozygous *MUTYH* c.934-2A>G variants (L130, L279, L522 and L239), wild-type *MUTYH* (L381 and L545), as well as from individuals harboring homozygous mutant *MUTYH* (L141 and L142) were assessed. No differences in cell viability were noted in the cell lines grown with or without supplemented magnesium over 96 hours. All reactions were performed in triplicate and results presented as mean ± standard deviation.

Supplementary Table 1. Pathogenic germline variants identified in proband

Gene name	Gene coordinates	Variant type	Nucleotide change	Protein change	dbSNP rsID	ClinVar ID
SLC12A3	16:56899326<br><br>16:56913130	Missense<br><br>Missense	c.179<br><br>c.1326	p.T60M<br><br>p.N442K*	rs371443644<br><br>rs775232139	101514<br><br>*nil*
MUTYH	1:45797760	Splicing	c.934-2	-	rs77542170	41766

*Predicted pathogenic based on multiple *in silico* predictors (see Supplementary Table 2)

Supplementary Table 2. Predicted functional impact of *SLC12A3* c.1326

Algorithm	Prediction score	Predicted functional impact
PROVEAN[22]	-5.47	Deleterious
SIFT[23]	0.001	Damaging
PolyPhen-2[24]	0.858	Damaging

Supplementary Table 3. List of lymphoblastoid cell lines used in the study

ID	MUTYH status	Individual information
L130	c.934-2A>G heterozygous	Proband
L522	c.934-2A>G heterozygous	Mother of proband
L381	Wild-type	Father of proband
L279	c.934-2A>G heterozygous	Sister 1 of proband
L545	Wild-type	Sister 2 of proband
L239	c.934-2A>G heterozygous	Patient with invasive ductal carcinoma of breast
L141	c.934-2A>G homozygous	Patient with thyroid and liver cancer
L142	c.721C>T homozygous	Patient with colorectal cancer
L258	Wild-type	Healthy donor 1
L259	Wild-type	Healthy donor 2
